# Effects of a bacteria-produced algicide on non-target marine invertebrate species

**DOI:** 10.1038/s41598-020-79814-w

**Published:** 2021-01-12

**Authors:** Victoria E. Simons, Kathryn J. Coyne, Mark E. Warner, Margaret M. Dolan, Jonathan H. Cohen

**Affiliations:** School of Marine Science and Policy, College of Earth, Ocean, and Environment, University of Delaware, Lewes, DE 19958 USA

**Keywords:** Marine biology, Ecology

## Abstract

Harmful algal blooms (HABs) affect both freshwater and marine systems. Laboratory experiments suggest an exudate produced by the bacterium *Shewanella* sp. IRI-160 could be used to prevent or mitigate dinoflagellate blooms; however, effects on non-target organisms are unknown. The algicide (IRI-160AA) was tested on various ontogenetic stages of the copepod *Acartia tonsa* (nauplii and adult copepodites), the blue crab *Callinectes sapidus* (zoea larvae and megalopa postlarvae), and the eastern oyster *Crassostrea virginica* (pediveliger larvae and adults). Mortality experiments with *A. tonsa* revealed that the 24-h LC50 was 13.4% v/v algicide for adult females and 5.96% for early-stage nauplii. For *C. sapidus*, the 24-h LC50 for first-stage zoeae was 16.8%; results were not significant for megalopae or oysters. Respiration rates for copepod nauplii increased in the 11% concentration, and in the 11% and 17% concentrations for crab zoeae; rates of later stages and oysters were unaffected. Activity level was affected for crab zoeae in the 1%, 11%, and 17% treatments, and for oyster pediveliger larvae at the 17% level. Activity of later stages and of adult copepods was unaffected. Smaller, non-target biota with higher surface to volume could be negatively impacted from IRI-160AA dosing, but overall the taxa and stages assayed were tolerant to the algicide at concentrations required for dinoflagellate mortality (EC50 =  ~ 1%).

## Introduction

Harmful algal blooms (HABs) are a world-wide phenomena that are increasing in number and intensity, leading to growing efforts to identify methods for prevention or mitigation of blooms^1–5^. With harmful algal blooms comes the bioavailability of harmful compounds these algae produce; in Delaware, USA, blooms of harmful algae including *Karlodinium veneficum* have been reported^6^, with *K. veneficum* producing karlotoxins responsible for hemolytic, cytotoxic, and ichthyotoxic effects^7^. These toxins create pores in cellular membranes that disrupt ion gradients, leading to lysis and ultimately fish kills^8,9^ and similarly negative effects on invertebrates^10,11^.

Natural compounds hold promise for combating HABs, provided that they minimally impact non-target organisms^1,12^. One potential approach to mitigate HABs is to apply naturally occurring species or the compounds they produce^1^. For example, Jeong et al.^13^ demonstrated that compounds extracted from certain seaweeds had algicidal effects on various red tide microalgae. Similarly, there are a growing number of bacterial isolates that have negative impacts on certain algae, some of which are irreversible and lead to cellular lysis^14–19^. In addition, algicidal bacteria and their compounds may be applied to HAB management, but more testing is needed to ensure possible negative environmental effects are avoided^20–22^.

*Shewanella* sp. IRI-160—a gammaproteobacteria isolated from Delaware waters –produces algicidal compounds. IRI-160 exudate (hereafter referred to as algicide, IRI-160AA, and IRI-160 algicide) negatively affected harmful dinoflagellates while exhibiting little to no negative effects on other algae; it appears to trigger cell cycle arrest and programmed cell death (PCD) in harmful dinoflagellates^23–27^. Thus, there is an interest in determining if IRI-160AA may mitigate blooms of harmful dinoflagellates in coastal waters. While the algicidal compound(s) have yet to be fully isolated^28^, an alternative approach is to determine how the whole bacterial exudate may affect non-target species^23–27^.

Any compound used to control or mitigate HABs may have unintended effects on higher trophic levels. Similar to other natural and anthropogenic stressors (e.g., temperature, salinity, oil, dispersant, etc.), these are manifested through either mortality^29–31^ or sublethal effects^32–36^. Lethal and sublethal assays with IRI-160AA (or a similar compound) and key metazoan taxa should help determine if food webs are altered by widescale application. A similar approach with IRI-160AA was recently tested at lower trophic levels: after exposing a planktonic community to the algicide, Tilney et al.^26^ noted an increase in heterotrophic protists and a bactivorous chrysophyte. After repeated IRI-160AA dosing, Grasso^27^ also noted a decline in dinoflagellates, while total algal biomass (recorded by fluorescence) was unchanged, thereby suggesting a community shift in phytoplankton that filled the void left by the dinoflagellates. Further, while some ciliate species also increased, community grazing rates either remained the same or increased after algicide application.

Beyond the microbial and microzooplankton communities, the effects of IRI-160AA on metazoans are unknown. Here, we tested IRI-160AA with three metazoan taxa. *Acartia tonsa* is a calanoid copepod that reaches lengths from 1 to 1.5 mm^37^ and is among the most abundant mesozooplankters in Mid-Atlantic estuaries, where they are critical phytoplankton grazers and food for larval and juvenile fish^38–40^. The blue crab, *Callinectes sapidus*, is a brachyuran crab found in Mid-Atlantic estuaries during its juvenile and adult life, while spending its larval period on the inner continental shelf^38,41^. This crab constitutes a large fishery along the United States Atlantic and Gulf coasts, making it both economically and ecologically important^41^. The eastern oyster, *Crassostrea virginica*, is a bivalve mollusk and an important Mid-Atlantic fishery, in addition to forming ecologically important oyster reefs^42^. *C. virginica* filter large quantities of sea water, and their larvae have low prey selectivity across different phytoplankton species^38^.

We established lethal algicide levels of IRI-160AA with these three common estuarine taxa, as well as sublethal algicidal effects through respiration and behavioral assays conducted in both light and dark to account for diel periodicity in their physiology and behavior.

## Results

### Lethal effects

**Figure 1:**
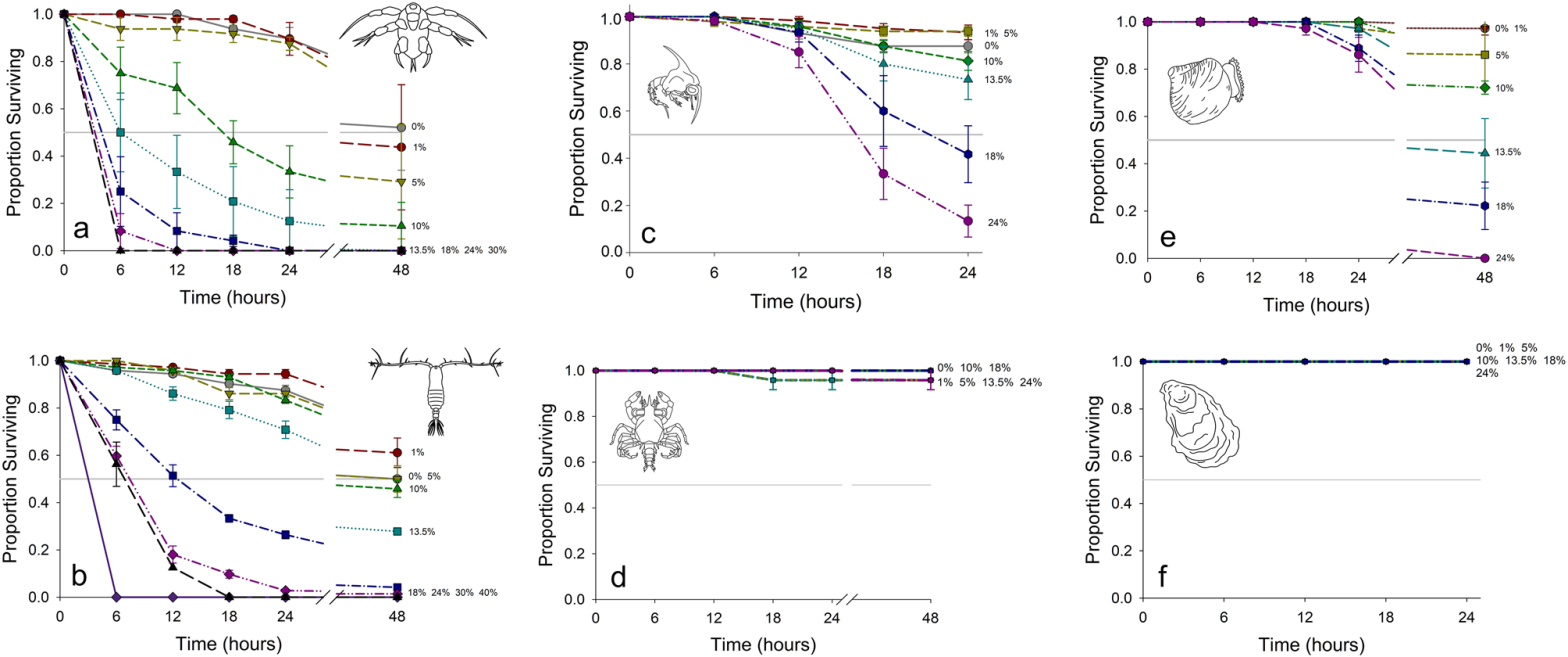
Survival of invertebrate taxa over time in different concentrations of IRI-160AA; the grey horizontal line marks 50% survival. (**a**) *A. tonsa* nauplii, (**b**) *A. tonsa* adult females, (**c**) *C. sapidus* zoeae, (**d**) *C. sapidus* megalopae, (**e**) *C. virginica* pediveligers, (**f**) *C. virginica* adults. See Table 1 for LC50s.

Species varied in their time-dependent mortality with algicide exposure (Tables 1, 2). *Acartia tonsa* nauplii had a 24-h LC50 of 5.96% v/v (*p* < 0.001, CI 4.54–7.29%) (Fig. 1a). For *A. tonsa* adult females, the LC50 was approximately two-fold higher at 12.2% (*p* < 0.001, 95% CI 10.6–13.9%) (Fig. 1b).

**Table 1:** LC50s (% v/v) for different organisms at different time points, along with their associated confidence intervals as determined from Probit analysis. (N. S. = Not significant, *p* > 0.05; “–“  Not tested).

	*A. tonsa*		*C. sapidus*		*C. virginica*	
	Nauplius	Adult female	Zoeae	Megalopae	Pediveliger	Adult
**48-h LC50**	1.09	N. S..	–	N. S.	10.9	–
Lower CI	0.497	N. S.	–	N. S.	8.56	–
Upper CI	1.78	N. S.	–	N. S.	13.2	–
*p*-value	0.027	N. S.	–	N. S.	< 0.001	–
**24-h LC50**	5.96	12.2	16.8	N. S.	N. S.	N. S.
Lower CI	4.54	10.6	13.5	N. S.	N. S.	N. S.
Upper CI	7.29	13.9	22.6	N. S.	N. S.	N. S.
*p*-value	< 0.001	< 0.001	< 0.001	N. S.	N. S.	N. S.
**18-h LC50**	8.62	14	26.2	N. S.	N. S.	N. S.
Lower CI	7.26	12.2	19.3	N. S.	N. S.	N. S.
Upper CI	9.85	16	49.4	N. S.	N. S.	N. S.
*p*-value	< 0.001	< 0.001	< 0.001	N. S.	N. S.	N. S.
**12-h LC50**	10.3	18	N. S.	N. S.	N. S.	N. S.
Lower CI	8.83	16.2	N. S.	N. S.	N. S.	N. S.
Upper CI	11.6	20.2	N. S.	N. S.	N. S.	N. S.
*p*-value	< 0.001	< 0.001	N. S.	N. S.	N. S.	N. S.
**6-h LC50**	N. S.	29.4	N. S.	N. S.	N. S.	N. S.
Lower CI	N. S.	25.6	N. S.	N. S.	N. S.	N. S.
Upper CI	N. S.	36.2	N. S.	N. S.	N. S.	N. S.
*p*-value	N. S.	< 0.001	N. S.	N. S.	N. S.	N. S.

**Table 2:** P-values from Gehan-Breslow survival analysis (*p* < 0.001 for all except *C. sapidus* megalopae and *C. virginica* adults, where *p* > 0.05) post-hoc analysis (Holm-Sidak). The left most column is concentration of algicide (% v/v). (– = Not tested; N. S. = Not significant, *p* > 0.05; * = *p* < 0.05; ** = *p* < 0.01, *** = *p* < 0.001).

[Algicide]	*A. tonsa*		*C. sapidus*		*C. virginica*	
	Nauplius	Adult Female	Zoeae	Megalopae	Pediveliger	**Adult**
1%	N. S.	N. S.	N. S.	N. S.	N. S.	N. S.
5%	**	N. S.	N. S.	N. S.	N. S.	N. S.
10%	***	N. S.	N. S.	N. S.	*	N. S.
13.5%	***	*	N. S.	N. S.	***	N. S.
15%	–	–	N. S.	–	–	–
18%	***	***	***	N. S.	***	N. S.
24%	***	***	***	N. S.	***	N. S.
30%	***	***	–	–	–	–
40%	–	***	–	–	–	-

*Callinectes sapidus* zoeae had a 24-h LC50 of approximately 16.8% v/v (*p* < 0.001, CI 13.5–22.6%) (Fig. 1c). Megalopae proved far more resistant to the algicide: no treatments had greater than 10% mortality, even after 48 h of exposure, thus we could not calculate an LC50 (Fig. 1d).

*Crassostrea virginica* pediveligers had less than 20% mortality through the 24-h time point. By 48 h, greater mortality allowed calculation of an LC50 of 10.9% v/v (*p* < 0.001, CI 8.56–13.2%) (Fig. 1e). For adult oysters, no individuals died during the experiments at any concentration or time point tested (Fig. 1f).

### Copepod sub-lethality

#### Activity

Total activity levels were unaffected in adult female *A. tonsa* exposed to all concentrations of algicide tested (one-way ANOVA on ranks, *p* = 0.952).

When activity was binned at 30 min intervals and separated into an initial light phase (L1), a dark phase (D), and a second light phase (L2) in order to account for diel activity rhythms, there still was no significant difference among treatments (one-way RMANOVA, *p* = 0.727). However, there was an overall difference between the initial light phase (L1) and final light phase (L2) (*p* = 0.039; Tukey post hoc, *p* = 0.041), with activity lower in the L2 phase. There was no interaction between treatment and time (Tukey post-hoc, *p* = 0.164).

**Figure 2:**
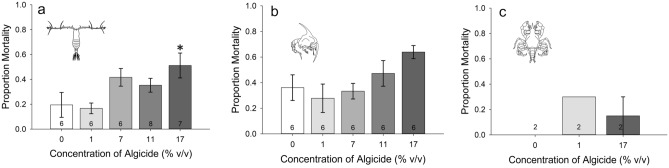
Mortality of invertebrate taxa during LAM experiments; mortality was assessed after 24 h in clean water following a prior 24-h algicide exposure. (**a**) Mortality of *A. tonsa* adult females. Only the 17% concentration had statistically higher mortality than the control (one-way ANOVA on Ranks, *p* = 0.012; Dunn’s test post-hoc, *p* = 0.040). Mortality of *C. sapidus* zoeae (**b**) and megalopae (**c**). Neither crab life stage had statistically significant mortality compared to the control for any algicide concentration tested (*p* > 0.05). (*p < 0.05).

Analyzing the proportion of dead individuals at the end of the activity experiments with *A. tonsa* adult females (i.e., after 24 h in clean water following a prior 24-h algicide exposure) revealed that only the 17% concentration had higher mortality relative to the 0% control (one-way ANOVA on Ranks, *p* = 0.012; Dunn’s test post-hoc, *p* = 0.040) (Fig. 2a).

#### Respiration

**Figure 3:**
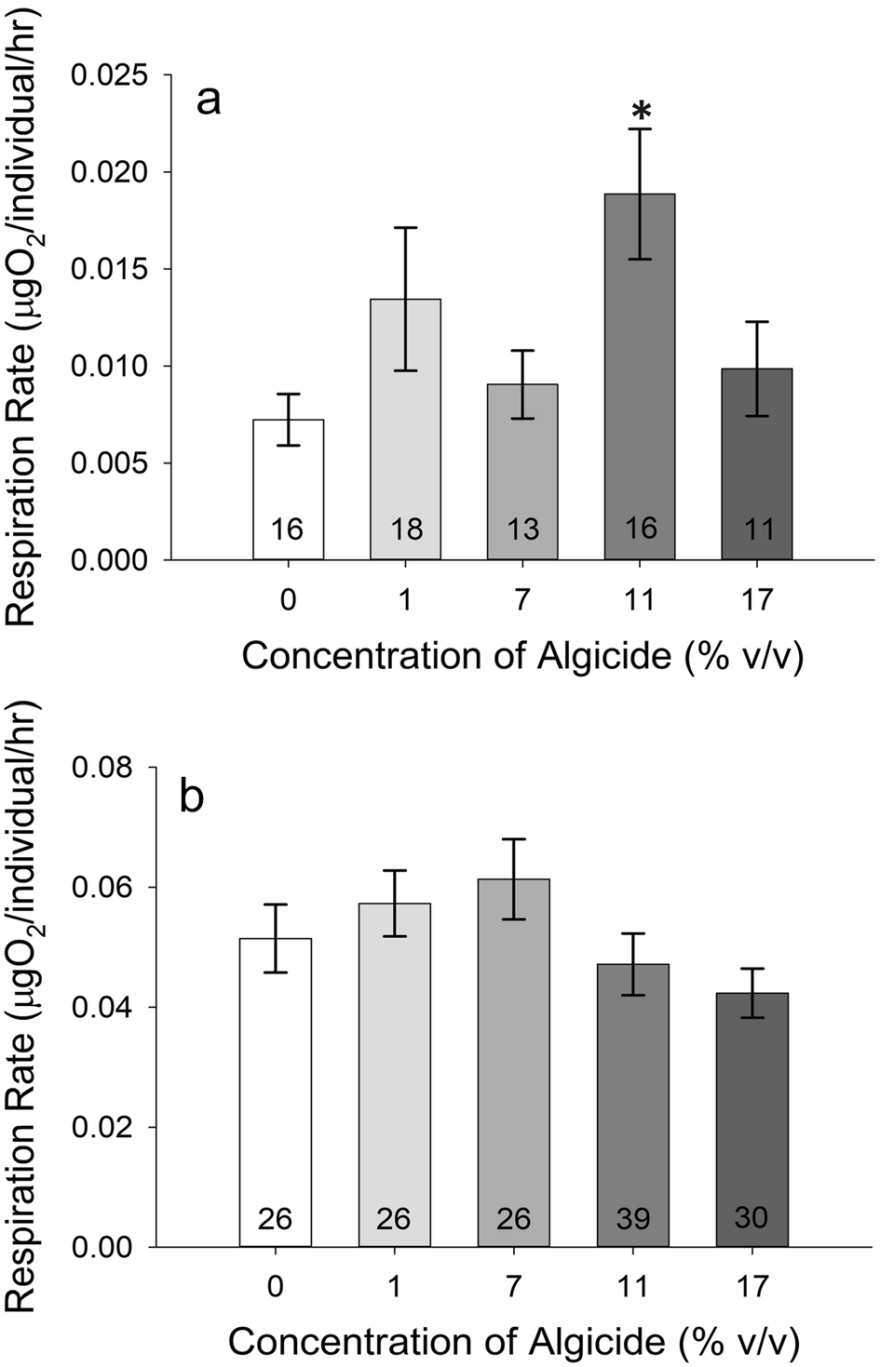
Respiration rates of *Acartia tonsa*; numbers on the bars represent sample size. (**a**) Respiration rate of nauplii *A. tonsa* as a function of algicide concentration. There was a statistically significant difference between the rates of the 11% animals and the 0% control animals (one-way ANOVA on Ranks, *p* = 0.039; Dunn’s Method post hoc, *p* = 0.012). (b) Respiration rate of adult female *A. tonsa* as a function of algicide concentration. (*p < 0.05).

Nauplii had higher respiration at the 11% concentration relative to the 0% control animals (one-way ANOVA on Ranks, *p* = 0.039; Dunn’s Method post hoc, *p* = 0.012), but not at any other concentration (Fig. 3a). Respiration rates of adult female *A. tonsa* were unaffected by 24-h algicide exposure (one-way ANOVA on Ranks, *p* = 0.115) (Fig. 3b).

### Crab sub-lethality

#### Activity

**Figure 4:**
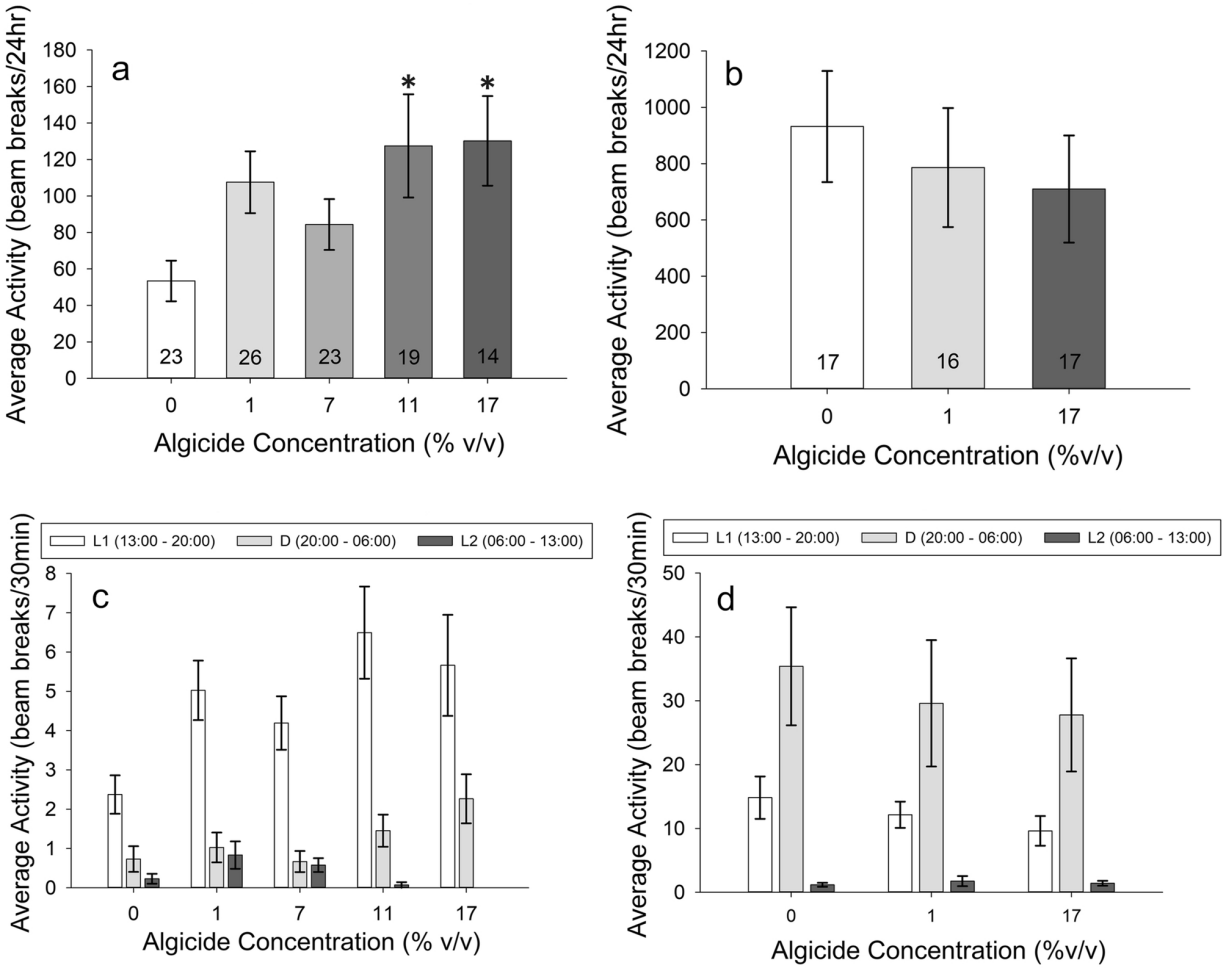
Activity levels of *Callinectes sapidus*; numbers on the bars represent sample size. Row 1: Activity level of *C. sapidus* zoeae (**a**) and megalopae (**b**) as a function of algicide concentration over a 24-h period. Animals were first pre-exposed to the listed concentrations of algicide for 24 h, then placed into tubes of algicide-free water for activity measurements over the subsequent 24 h. Row 2: Data from (**a**) and (**b**) broken down into activity per 30 min and separated by time of day for zoeae (**c**) and megalopae (**d**). Notice the scale differences among the graphs. (*p < 0.05).

Crab zoeae activity levels differed among treatments (one-way ANOVA, *p* = 0.020). While average values for all treatments exceeded the 0% controls, only at 11% and 17% algicide were these differences significant (pairwise Holm-Sidak post hoc, *p* = 0.044, *p* = 0.049, respectively) (Fig. 4a).When analyzed by light:dark phase (Fig. 4c), there was higher activity in the 1%, 11%, and 17% treatments versus the control during the L1 phase only for the zoeae (one-way RMANOVA, *p* < 0.001 for interaction; Tukey post hoc, *p* = 0.02, *p* < 0.001, *p* = 0.013 respectively). There were no other differences between any of the treatments. Differences disappeared during the dark phase (D) (*p* = 0.994 for 1%, 0.429 for 11%, and 0.062 for 17%) and during L2 (*p* = 0.79 for 1%, 0.986 for 11%, and 0.959 for 17%). Also of note was a difference between the two light phases and between the L1 and D phases for all concentrations, and only a difference between the D and L2 phases for the 11% and 17% treatments (*p* < 0.001 for all) (Fig. 4c). Overall, activity decreased with time. There were no significant differences in the proportion of dead individuals at the end of the LAM experiments for *C. sapidus* zoeae for any concentration (*p* = 0.058) (Fig. 2b).

There was no significant difference in activity among treatments for megalopae (one-way ANOVA on Ranks, *p* = 0.737) (Fig. 4b). There was also no significant difference among treatments for the megalopae when analyzed by light phase (one-way RMANOVA, *p* = 0.694) (Fig. 4d). However, there was an overall difference among the three light phases (*p* < 0.001; Tukey post hoc, *p* < 0.001 for all three comparisons). Activity was highest in the D phase, followed by the L1 phase, and was lowest in the L2 phase. There was no interaction between treatment and time for the megalopae (*p* = 0.773) (Fig. 4d). There was no difference among treatments for the proportion of individuals dead at the end of the activity experiments for megalopae (*p* = 0.4) (Fig. 2c).

#### Respiration

**Figure 5:**
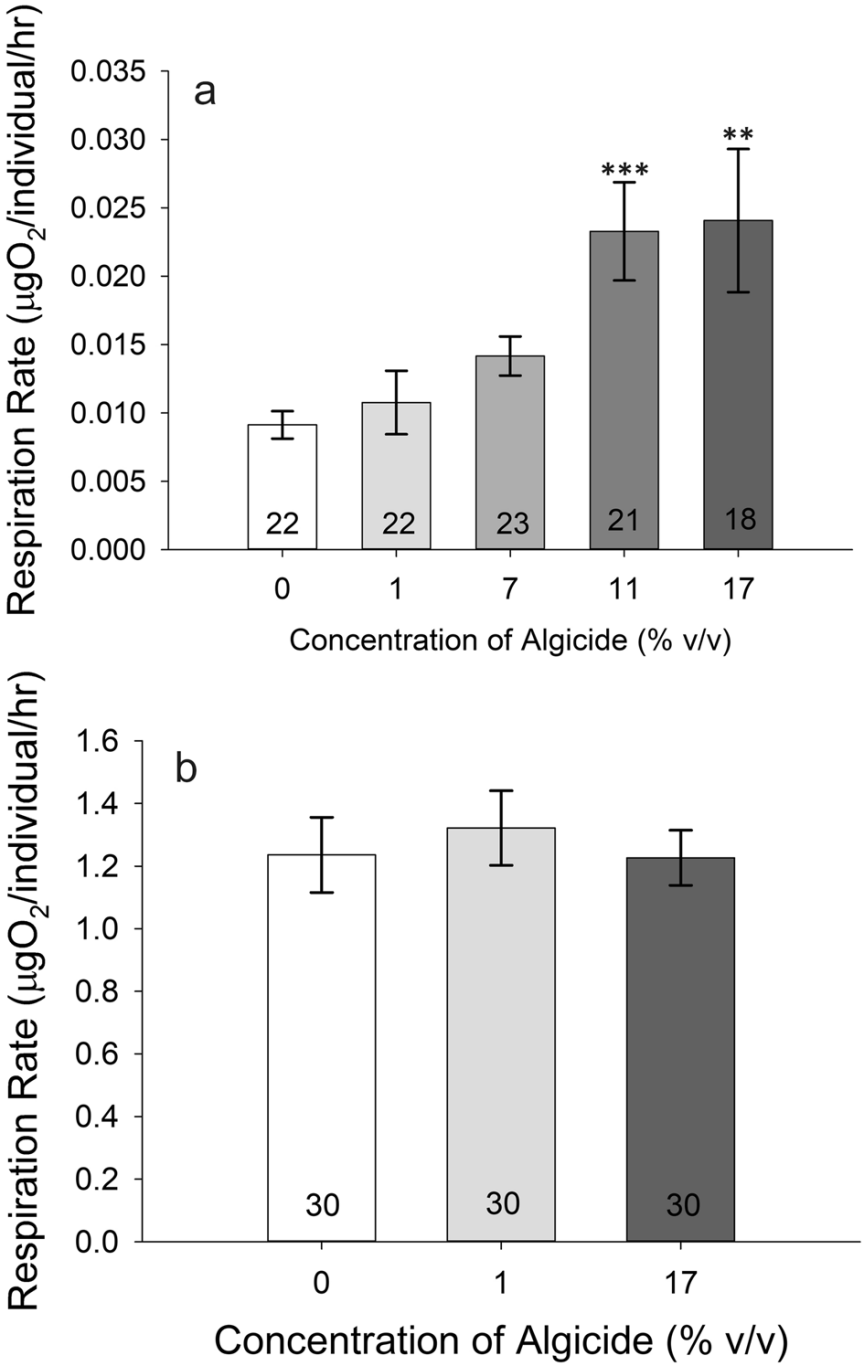
Respiration rates of *Callinectes sapidus*; numbers on the bars represent sample size. (**a**) Respiration rate of *C. sapidus* zoeae as a function of algicide concentration. Asterisks mark rates for algicide concentrations that are significantly different from the 0% control animals. (**b**) Respiration rate of *C. sapidus* megalopae as a function of algicide concentration. (**p < 0.01; *** = *p* < 0.001).

Crab zoeae had higher respiration rates at the 11% and 17% algicide concentrations relative to the 0% control, but not at lower concentrations (one-way ANOVA on Ranks, *p* < 0.001; Dunn’s Method post hoc, *p* < 0.001 for 11% and *p* = 0.007 for 17%) (Fig. 5a). Megalopae respiration rate was unaffected by algicide at either concentration tested (1% and 17%; one-way ANOVA on Ranks, *p* = 0.396) (Fig. 5b).

#### Metamorphosis

Time to metamorphosis (TTM) of megalopae to the first juvenile crab stage was unaffected by 24-h algicide exposure at 0%, 1%, and 17% concentrations (Kaplan–Meier Survival Analysis; Gehan-Breslow, *p* = 0.564) (Supplementary Fig. S1). The median TTM for all three concentrations was 72 h.

#### Abdomen pumping and grooming

We examined both pumping and grooming of the egg mass in ovigerous female *C. sapidus* when exposed to homogenized eggs (which provides a natural cue eliciting these behaviors) with and without algicide. Pumping and grooming did not differ with algicide exposure (χ^2^ test, pumping, *p* = 0.29; grooming, *p* = 0.441) (Supplementary Fig. S2). For the number of pumps (n = 9 crabs) and time spent grooming (n = 12 crabs), there were no significant differences between any algicide treatment and the saltwater with homogenized eggs (SW + HE) control (*p* > 0.05) (Supplementary Fig. S2).

### Oyster sub-lethality

#### Swimming activity

**Figure 6:**
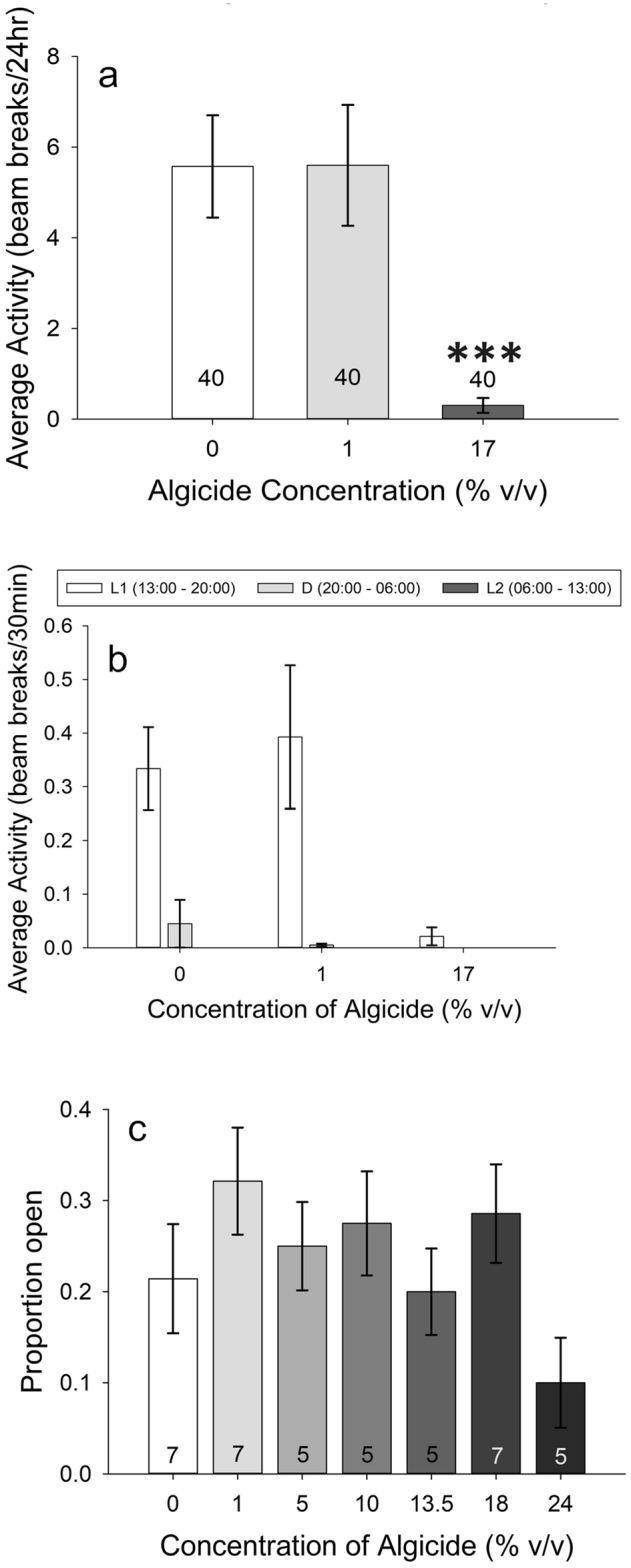
Activity levels of *Crassostrea virginica*; numbers on the bars represent sample size. (**a**) Activity level of *C. virginica* larvae as a function of algicide concentration over a 24-h period. Animals were first pre-exposed to the listed concentrations of algicide for 24 h, then placed into tubes of algicide-free water for activity measurements over the subsequent 24 h. (**b**) Data from (**a**) broken down into activity per 30 min and separated by time of day. (**c**) Adult *C. virginica* activity, given as proportion of individuals open. Animals were assessed in containers in pairs, creating a set of containers where the proportion open was either 0, 0.5, or 1. These were checked at 4 time points and scored, data were averaged across time points to calculate an average proportion open for the container, then containers were averaged and plotted (n values are given on each bar). (***p < 0.001).

*C. virginica* pediveliger swimming activity dropped significantly at the 17% concentration relative to the 0% control animals, but not at the 1% concentration (one-way ANOVA on Ranks, *p* < 0.001) (Fig. 6a).

When analyzed by light:dark phase, there was an interaction between light:dark phase and treatment (one-way RMANOVA, *p* < 0.001) (Fig. 6b). Within the L1 phase, swimming activity was reduced in the 17% treatment relative to 0% controls (Tukey post hoc, *p* < 0.001), but this difference disappeared in the dark (*p* = 0.681) and L2 (*p* = 1) phases. There was a significant difference between L1 and L2 and between L1 and D for both the 0% and the 1% algicide treatment (*p* < 0.001 for all), but not for the 17% (*p* = 0.913) (Fig. 6b). Overall, *C. virginica* pediveliger activity decreased with time.

**Figure 7:**
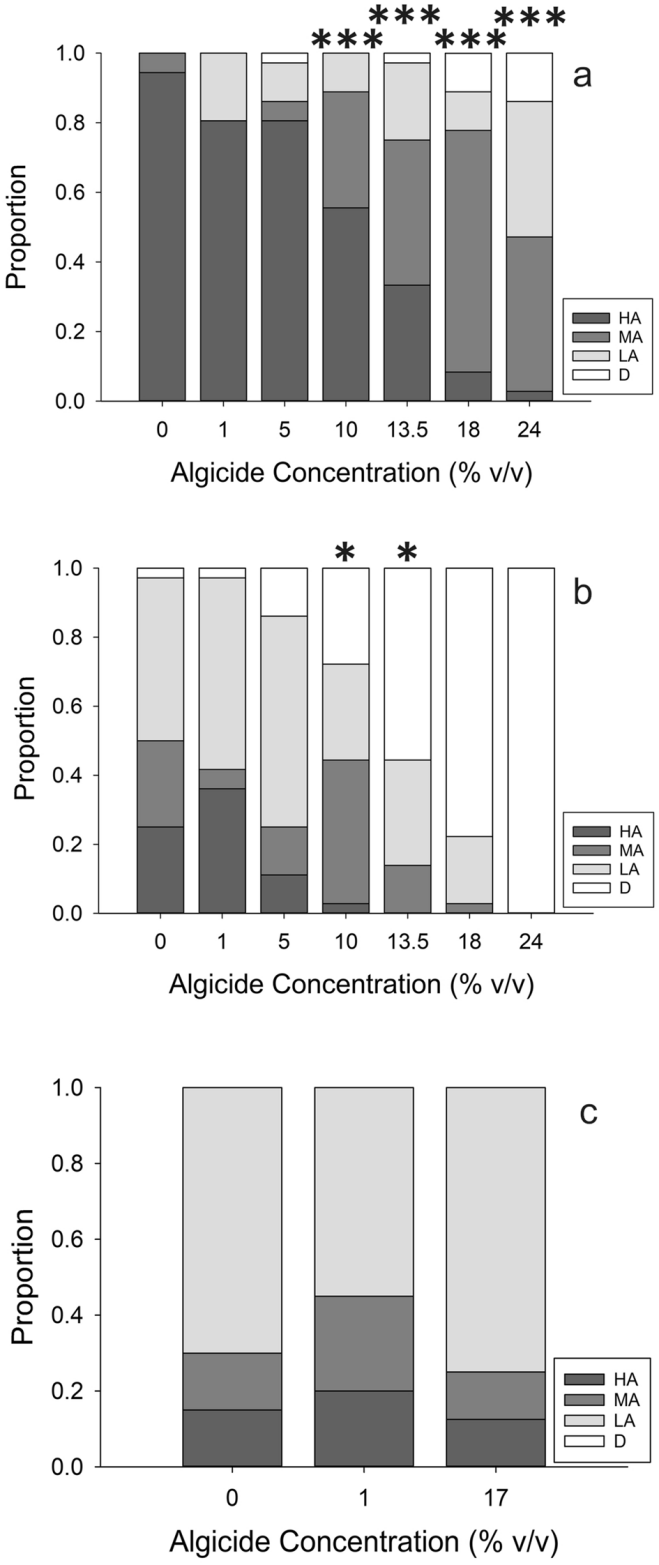
Activity of *C. virginica* larvae during the mortality experiments. Activity was scored at the beginning of the experiment (where all animals were scored High Activity), then at 24 (**a**) and 48 (**b**) hours. The 18% and 24% concentrations were unable to be tested at 48-h due to high mortality. Concentrations 10% and higher lead to changes in activity. Activity from the end of the LAM experiments is given in plot (**c**). There were no differences in activity at the end of the LAMs. (*p < 0.05; ***p < 0.001).

We assessed additional oyster pediveliger activity during the mortality assay (i.e., while still exposed to algicide, and in contrast to locomotor activity monitor [LAM] experiments conducted in clean water after algicide exposures). For this assessment (which scored animals in the plates as exhibiting High, Medium, and Low activity at 24 and 48 h; see Methods for details), there was a significant difference among algicide treatments at both the 24- and 48-h time points (χ^2^ test, *p* < 0.001 for each time point) (Fig. 7a,b). There was no significant difference among treatments in the same activity assessment at the end of the LAM experiment, (χ^2^ test, *p* = 0.401) (Fig. 7c). When activity was binned as either active (swimming, velum extended) or inactive (sitting on the bottom, velum retracted), both the 24- and 48-h assessments were significantly different (χ^2^ test , *p* < 0.001 for 24 h and *p* = 0.002 for 48 h), while activity among treatments at the end of the LAM experiments were indistinguishable (*p* = 0.646). To determine which treatments were different from the controls in 24- and 48-h assays, multiple pairwise Fisher Exact Tests were run to compare each treatment to the 0% for both time points. At 24-h, the 10%, 13.5%, 18%, and 24% were all different from the control (*p* < 0.001 for all). At 48-h, the 10% and 13.5% were both different from the control (*p* = 0.034 and 0.043); the 18 and 24% were not compared due to high mortality. Overall, there was a trend of fewer animals marked as High activity with time, and with increasing concentration of IRI-160AA.

For adult *C. virginica*, there was no difference in the proportion of individuals with open shell valves based on treatment, time, or their interaction (*p* = 0.582 for treatment, 0.160 for time, and 0.492 for treatment *x* time) (Fig. 6c). However, in the 24% treatment at both the 18- and 24-h time points, no individual had opened shell valves, which was not seen in any other concentration, including controls.

#### Respiration

*Crassostrea virginica* pediveliger respiration rates did not differ among the algicide concentrations (one-way ANOVA on Ranks, *p* = 0.093). Mean respiration rates were 0.0072 µgO^2^/individual/hour for controls, 0.0066 µgO^2^/individual/hour for the 1% treatment, and 0.0046 µgO^2^/individual/hour for the 17% treatment.

#### Feeding

Clearance rates (CR) of adult *C. virginica* were similar across the different strains (wild-type and hatchery) and algicide concentrations (three-way ANOVA, *p* > 0.05). There was a difference in CR based on exposure time (initial 6-h period, 0–6, versus second 6-h period, 6–12), independent of other factors (three-way ANOVA, *p* < 0.001). CR in the 0–6 interval were higher than rates in the 6–12 interval (Supplementary Fig. S3).

## Discussion

IRI-160AA is being investigated as an algicide for controlling and mitigating regularly occurring harmful dinoflagellate blooms in coastal ecosystems. Here, we evaluated its effects on non-target invertebrates. All taxa assayed had LC50s above the effective algicide concentration needed for controlling dinoflagellate growth (EC50 ≈ 1% v/v.)^27^. For most taxa tested, the LC50 was a factor of 10 or greater above the dinoflagellate EC50. The most sensitive species/stage tested was *A. tonsa* copepod nauplii, with an LC50 ~ fivefold higher than the dinoflagellate EC50. Importantly, the 1% treatments had very high survival at 24 h for all taxa tested – showing greater than 85% survival – and were not significantly different from the control.

**Figure 8:**
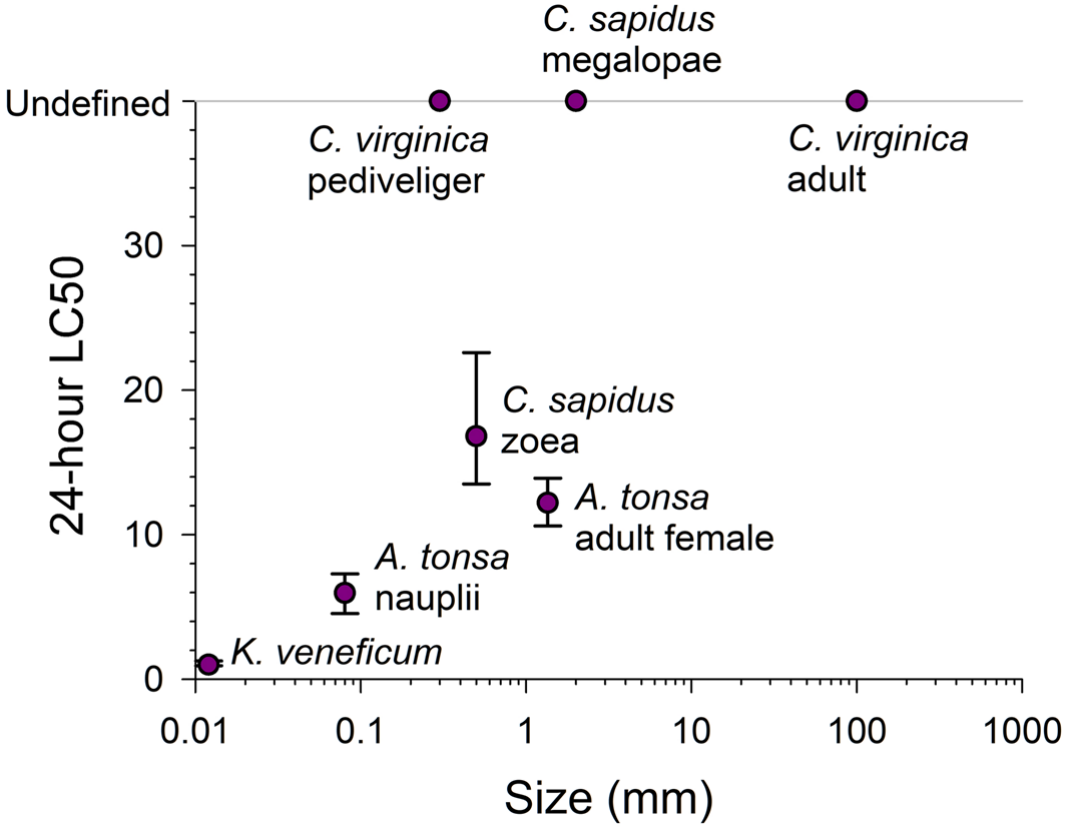
Size and 24-h LC50 for various animal species, as well as the EC50 for the harmful dinoflagellate *Karlodinium veneficum*. Error bars are 95% CIs for LC50s. The grey line at the top is used to plot animals where a 24-h LC50 was not calculated. As size increases, generally so does LC50.

For our mortality assays in general, the smaller, earlier life stages were more sensitive to the algicide than the larger, later life stages (Fig. 8). Similar effects of stage/size, or perhaps more directly surface area to volume^43^, have been reported for invertebrate mortality and sub-lethality in response to other contaminants such as pesticides^44^, dispersants^33^, and certain metals^45^. Given their ability to control tissue exposure by closing their shells, adult and larval oysters are unique among the taxa we tested. Such behavior helps explain why oyster pediveligers, despite being young and small, were more resistant to the algicide at 24-h compared to larger, older organisms like adult copepods. A similar point was reported by Tilney et al.^26^, where dinoflagellates with greater membrane exposure (e.g., athecate dinoflagellates) were more sensitive to IRI-160AA.

Beyond mortality, stressors can both positively and negatively affect invertebrate activity and physiology. Cohen et al.^33^ noted copepod swimming was negatively affected by both dispersed and undispersed crude oil. Porter and Brietburg^34^ recorded how Eastern oyster behavior changed in response to diel-cycling hypoxia; specifically, under severe hypoxic conditions the animals closed their shells, and reopened once waters became normoxic, indicating that these animals respond to environmental stressors with effective coping mechanisms. Forward et al.^35^ documented how environmental factors like humic acids and ammonia from estuaries help trigger or hinder blue crab molting from megalopae to first crabs, indicating the importance of environmental factors to their life history.

In the present study, mortality results suggested copepods are more vulnerable to algicide than are crab zoeae and oyster pediveligers. However, we observed greater sub-lethal effects in crab zoeae and oyster pediveligers than in copepods. Adult female copepods showed no signs of sub-lethal effects (activity or respiration) at any of the algicide concentrations. Copepod nauplii were not assayed for activity level due to their small size; however, their respiration rate was elevated in the 11% treatment. This concentration is above their calculated ~ 6% LC50. Accordingly, for individuals that manage to survive initial exposure at the higher dosing concentrations that would be required in a field setting, effects after 24-h of algicide exposure may persist in clean conditions. Sublethal effects were more pronounced in both crab zoeae and oyster pediveligers, but again these were mostly limited to higher algicide concentrations that would more likely occur over limited areas during initial algicide dosing of a bloom.

Irrespective of time of day and tide, first-stage blue crab zoea larvae normally swim in order to remain in surface waters and move from estuaries to the coastal ocean^46^, thus no effect of time of day was expected. When activity was split by light phase, increased activity due to the algicide was limited to the first light phase; there was no difference in any treatment compared to the control during the dark phase and second light phase. Additionally, respiration rate did not increase in the 1% concentration, while activity and respiration rates were elevated in the 11% and 17% concentrations, suggesting that elevated activity was coupled with higher metabolic rates at these higher concentrations. Unlike zoeae, there were no sublethal effects in postalarval megalopae with algicide exposure. Interestingly, Forward et al.^35^ documented inhibited TTM with ammonium concentrations ≥ 25 µM, and IRI-160AA contains ammonium > 60 µM (unpublished data). However, Forward et al.^35^ constantly exposed animals to ammonium, while our results are from animals exposed to algicide for 24 h and then assessed in clean sea water.

In the first light phase, oyster pediveliger larvae activity strongly decreased in the 17% LAM treatment, and similar to the crab zoea larvae, this response disappeared in the dark phase and second light phase; this was measured in clean water after algicide exposure. When measured during algicide exposure, oyster pediveliger activity declined at concentrations ≥ 10%. In both assays, oyster pediveligers reduced swimming activity and closed their shell valves, thereby isolating their tissue and reducing exposure to the algicide. This is consistent with how juvenile/adult bivalves adjust shell gaping in response to other environmental factors^34,47,48^. However, while adult oysters minimized their shell gape when exposed to high algicide, this had no effect on their clearance rate.

Collectively, lethal and sublethal assays suggest earlier, smaller life stages are more sensitive to this algicide than later, larger stages. Nevertheless, algicide effects were minimal or absent at the algicide concentration required to inhibit dinoflagellate growth (EC50 ≈ 1% v/v)^27^. It is likely that local algicide application for HAB control and mitigation will require concentrations > 1% in order to reach this concentration over larger spatial scales. The highest algicide concentration we tested was 40%; high concentrations resulted in a range of effects, but these were highly species and stage dependent. Future modeling work should examine water residence time in potential application areas to assess what field concentrations animals would likely be exposed to, and how long those exposures would last.

More generally, isolated algicidal compounds such as IRI-160AA are promising in their role of controlling harmful dinoflagellate blooms. While there may be risks to metazoans at the site of application where locally high concentrations would be experienced, these effects are limited. Given the substantial negative impacts of HABs across higher trophic levels^10,11,47,51,58^, the risk of direct exposure of metazoans to algicide during control seems tolerable. This should be confirmed in mesocosm studies where dinoflagellates and metazoans can be exposed to algicide simultaneously.

## Methods

### Algicide preparation

Four batches of algicide were used for experiments, labeled Batch 3, Batch 4–5–6, Batch 7, and Batch 8, following methods used by Grasso^27^. For each batch, a single colony of *Shewanella* sp. IRI-160 was transferred from a modified LM medium plate to liquid LM medium for overnight growth, then inoculated into f/2 with 0.05% casamino acids and incubated for 10 days at room temperature with bubbling. Bacteria and other compounds greater than 60 kDa in size were filtered out using a HemoFlow HF80S 60 kDa dialysis cartridge (Fresenius Medical Care, Waltham, MA), creating a batch of sterile filtered exudate referred to as IRI-160AA. Samples of the algicide were diluted with ultrapure water, then total nitrogen (TN) was measured with a TOC-V total organic carbon analyzer equipped with a Total Nitrogen Measuring Unit (Shimadzu Corp., Kyoto, Japan). The algicide has approximately 5.02 mg/L TN. The 24-h EC50 for *K. veneficum* differed among batches but was always close to 1% (actual EC50s ranged from 0.93% in Batch 4–5–6 to 1.5% in Batch 3), thus a 1% concentration of the algicide was included in all invertebrate assays^27^. Animals were also exposed to a media control to ensure mortality was due to the algicide.

### Statistical analyses

For all statistics, data were analyzed using Shapiro–Wilk normality tests and Brown-Forsythe equal variance tests. If they failed either, data were transformed and reanalyzed. If transformed data passed both tests, then analysis proceeded. If neither log or square-root transformed data passed both normality and equal variance tests, then a non-parametric test was run if possible. Specific details on statistical analyses are provided in each section below.

### Copepod mortality

Mortality experiments followed established methods for determining acute toxicity in aquatic animals^30,31,33,49^. For *A. tonsa* adults, we collected animals in Fall of 2018 after sunset near the mouth of the Broadkill River (Delaware, USA) using a plankton net. Cod ends were diluted and maintained in field collected seawater with ambient food at room temperature (~ 20 °C) until use in experiments. Adults were filtered out of the bulk collection with a 500-μm mesh, then sorted for adult females. We transferred one adult female (n = 24 for 40%, 48 for 30%, and 72 for all other concentrations) into each well of a 12-well plate containing 5 mL of test solution; test solutions included a seawater control (0%); algicide mixtures prepared from Batch 3 of the IRI-160AA in 20 psu, 0.2 μm-filtered sea water collected from Indian River Inlet, DE, USA (FSW) (1%, 5%, 10%, 13.5%, 18%, 24%, 30%, and 40% v/v); and a 24% media solution as a media control. The plates were incubated at 25 °C in low-light (~ 2.37 × 10^13^ photons cm^-2^ s^−1^) on a 14:10 h day:night cycle for 48 h. Every 6 h for the first 24 h, and again at 48 h, we counted the number alive and dead.

For *A. tonsa* nauplii, adult females and males were placed in two 1 L beakers at room temperature with a 150-μm mesh placed several centimeters off the bottom (to prevent egg cannibalism), a slow bubbler (~ 2 small bubbles s^−1^), and ambient seawater diluted with 20 psu FSW until the water was mostly clear. Adults were allowed to mate in the beaker for approximately 24 h, after which we removed the mesh, thus removing the adults and leaving behind any nauplii and eggs. After another 24 h, the contents of the beakers were poured through a 20-μm mesh, and we extracted the nauplii and placed them into experimental treatments (0% seawater control, algicide at 1%, 5%, 10%, 13.5%, 18%, 24%, and 30% v/v concentrations, plus a 24% media control; n = 48 animals for all concentrations) following the procedure outlined above for the adult female copepods. This experiment was conducted three times; the first two mortality experiments used Batch 3 of the IRI-160AA, and the third mortality experiment used Batch 8.

From the data collected, we generated a Probit model^50^ and obtained a 24-h LC50. Another approach looks at mortality over several time points in order to generate a time series of survival (e.g., Robineau et al.^51^, Keller et al.^52^). This also allows the generation of an LC50 at several time points (e.g., 6, 12, 18, and 24 h), which can better inform how a certain animal may survive over time. We used SigmaPlot to generate graphs of survival over time, and R statistical software^53^ and the R package *ecotoxicology*^54^ for generating and graphing the Probit model and running a χ^2^ test to evaluate the model.

### Crab mortality

We conducted mortality experiments for the blue crab (*Callinectes sapidus*) in larval (Z1-stage zoeae) and postlarval (megalopae) stages in a similar manner to mortality experiments with *Acartia tonsa*. We collected ovigerous female blue crabs during the Summer of 2018 by dip net and drop net at sunset from the Delaware Bay (similar to methods used by Kernehan^55^) in Cape Henlopen State Park and maintained them in a recirculating water tray containing filtered ambient seawater (~ 30 psu) at room temperature. We staged egg masses every few days^55^, and females predicted to hatch within ~ 3 days were moved to 7-gallon buckets in a 25 °C incubator containing ~ 30 psu sea water and a bubbler. Zoea larvae (Z1-stage) hatched from these females were kept in large finger bowls with 30 psu sea water at room temperature and were fed lab-reared rotifers (*Brachionus rotundiformis*, Reed Mariculture). These animals became subjects for mortality and sub-lethal experiments within approximately a day of hatching. Four experiments were conducted; three mortality experiments used Batch 4–5–6 of the IRI-160AA, while the fourth experiment (24 individuals for each concentration) used Batch 7.

Megalopae were collected by plankton net set on rising tides at night during the Summer and Fall of 2018. They were maintained in large finger bowls at room temperature and fed with *Artemia* nauplii and went into experiments within a few days of collection. Only megalopae in intermolt based on morphology^56^ were used in experiments. Megalopae experiments used Batch 3 of the IRI-160AA.

Both zoeae and megalopae were exposed to 1%, 5%, 10%, 13.5%, 18%, and 24% algicide concentrations, plus a 0% seawater control and a 24% media control (n = 84 animals for the 0% concentration and 60 for all other concentrations for zoeae, and n = 24 animals for megalopae for all concentrations). Animals were incubated at 25 °C under low-light (~ 2.37 × 10^13^ photons cm^-2^ s^-1^) on a 14:10 light:dark cycle for the duration of experiments. We checked on zoeae and megalopae every 6 h for 24 h; megalopae were checked at an additional 48-h time point.

### Oyster mortality

Oyster larvae (eyed pediveligers of *Crassostrea virginica*) were provided by University of Maryland’s Horn Point Laboratory. Animals were maintained on a damp coffee filter in a sealed plastic container on ice during transport, then released into room-temperature fingerbowls containing 20 psu water and fed a locally-isolated alga (*Storeatula major*) at room temperature. Experiments occurred in similar fashion to those conducted on *Acartia tonsa* and *Callinectes sapidus*. Larvae were assayed in 12-well plates (n = 36 animals for all concentrations). Animals were exposed to 1%, 5%, 10%, 13.5%, 18%, and 24% algicide concentrations, plus a 0% seawater control, and 24% media control. Animals were incubated at 25 °C under a 14:10 light:dark cycle for the duration of experiments. Survival was evaluated every 6 h for 24 h and again at 48 h. Larvae were additionally examined at the start of the experiment and at the 24- and 48-h time points for an activity assay. These experiments used Batch 3 of the IRI-160AA.

Wild-type adult *C. virginica* were collected from the Delaware Bay near the University of Delaware Lewes Campus, while Haskins-disease-resistant strain individuals were collected from aquaculture cages maintained by the Delaware Center for the Inland Bays. On the first day, individuals were cleaned with a wire brush, and divided into two buckets containing approximately 10 L of 20 psu seawater and were fed *Isochrysis galbana* (~ 100,000 cells L^−1^). On the second day the water was changed and they were again fed. On the third day, water was changed and animals were not fed. On the fourth day, individuals were removed from the buckets, dried with a paper towel, labeled with permanent marker, and placed in pairs into forty-one 1 L plastic containers containing 1 L of various algicide solutions: 0%, 1%, 5%, 10%, 13.5%, 18%, and 24% (n = 28 for 0%, 22 for 1% and 18%, and 20 for all other concentrations). Individuals were checked every 6 h for 24 h and assessed if they were alive or dead. Closed individuals were assumed to be alive. If open individuals were observed, we gently tapped on the container to see if the individual shut its shell; animals that responded to this stimulus were marked as alive. Only animals that did not respond to repeated stimuli were scored as dead. Proportion surviving was compared across algicide concentration and strain. These experiments all used Batch 8 of the IRI-160AA.

### Copepod sub-lethality

#### Respiration

We conducted respiration experiments on *A. tonsa* adult females and young nauplii in a 24-well microplate respirometer (Loligo Systems). First, we sorted animals into fingerbowls containing 100 mL of their respective algicide concentrations. After 24 h of algicide exposure, we removed animals via pipette and put one animal into each well of the respirometer plate (200 μL wells for adult females and 80 μL for nauplii) filled with 0.2 μm filtered FSW, then sealed the plate with Parafilm and a weight. Each experiment also had 4 to 6 wells with only FSW to calculate background oxygen consumption. The experiment occurred in darkness within a 25 °C incubator at night and lasted several hours (n = 26–39 animals for adult females, 11–18 for nauplii). Oxygen concentrations in each well were recorded every minute. At the end of the experiment, respiration rates were calculated in R statistical software using the *respR* package^57^ over a period of time when the animals were still in independent respiration, and a one-way ANOVA on ranks in SigmaPlot (Systat Software, San Jose, CA) compared treatments. Experiments with adult females used Batch 3 of IRI-160AA, while nauplii experiments used Batch 8.

#### Activity

Experiments determining effects on swimming activity utilized Locomotor Activity Monitors (LAMs; TriKinetics). Three beams of infrared light cross a 3 mL test tube containing an animal and register when the animal crosses the beams. We sorted batches of adult female *A. tonsa* into fingerbowls containing different algicide treatments. Animals were incubated at 25 °C in low-light conditions (~ 2.37 × 10^13^ photons cm^−2^ s^−1^) for 24 h on a 11:13-h light:dark cycle. Animals were pipetted into plastic test tubes (one animal per tube) containing ~ 3 mL of FSW, which then went into the LAMs (n = 21–36 animals). The experiment lasted 24 h with beam breaks summed at one-minute intervals, allowing the data to be analyzed wholly for the 24-h period as well as across different light phases to account for light:dark mediated activity rhythms. Experiments started in the afternoon and ran overnight, creating an initial light phase (L1), a dark phase (D), and a second light phase (L2). Comparing treatments across the entire time period was done using a one-way ANOVA on ranks, while analyzing the data based on the different light phases was performed via a one-way repeated-measures ANOVA. Additionally, at the end of the LAM activity experiments we collected the individuals and noted mortality. This data was analyzed via a one-way ANOVA on ranks. Copepod activity experiments used Batch 3 of the IRI-160AA. Nauplii were too small to generate a reliable signal in the LAMs and were not used in these experiments.

### Crab sub-lethality

#### Respiration

Respiration experiments followed methods described for *A. tonsa* above and involved zoeae and megalopae. A one-way ANOVA on ranks was calculated using the data for each life stage. The first four zoeae experiments used Batch 4–5-6 of IRI-160AA, while the last two experiments used Batch 7. Megalopae experiments all used Batch 3.

#### Activity

Activity level experiments followed methods described for *A. tonsa* above and involved zoeae and megalopae. The 24-h data were analyzed using a one-way ANOVA on square root transformed data for zoeae, and a one-way ANOVA on ranks for megalopae. The data broken down by light phase were analyzed via one-way repeated measures ANOVA on log-transformed data for both zoeae and megalopae. These experiments all used Batch 3 of IRI-160AA.

At the end of experiments we collected the individuals and noted mortality. This data was analyzed via a one-way ANOVA for zoeae and a one-way ANOVA on ranks for the megalopae.

#### Metamorphosis

We sorted megalopae into finger bowls containing 100 mL of filtered estuary water with different concentrations of the IRI-160AA algicide (0%, 1%, and 17% v/v). After 24-h of exposure, we sorted animals into 12-well plates containing FSW (n = 60 individuals for each treatment). Water was changed daily, and animals were fed freshly hatched *Artemia* daily. Every 12 h, we counted how many megalopae had molted into first crabs until most had metamorphosed (5.5 days) and used a Kaplan–Meier Survival Analysis with a Gehan-Breslow test to determine if there was a difference in time to metamorphosis (TTM) across treatments. These experiments used Batch 3 of the IRI-160AA.

#### Abdomen Pumping and Grooming

Crabs with egg masses were collected from the Delaware Bay near Lewes, DE and separated into numbered baskets and maintained in a flow-through sea water table. They were fed thawed squid (*Loligo opalescens)* every day, and eggs were photographed every two to three days under a dissecting scope until they reached ~ 6 days until hatching (i.e., late-stage sensu Tankersley et al.)^36^. Homogenized egg water (seawater plus homogenized eggs, designated SW + HE, ~ 20 eggs mL^−1^) was utilized to induce pumping and grooming behavior and made according to Tankersley et al.^36^.

Ovigerous females were exposed to several sub-lethal concentrations of algicide combined with the homogenized egg solution and monitored for pumping and grooming behavior. Test solutions were diluted to 1.5 L with filtered 30 psu seawater, and 3.75 mL aliquot of a pre-prepared homogenized egg solution was added to achieve a final concentration of ~ 20 eggs/mL. These experiments used Batch 4–5–6, Batch 7, and Batch 3 of the IRI-160AA.

Between three and six crabs were tested at a time, and all crabs were staged the day of the experiment to verify that their eggs were no more than six days from hatching. All experiments were performed under dim red light to reduce disturbance. Each crab was tested in every treatment. A crab was placed into a translucent container (20.1 × 16.5 × 11.4 cm) with a given treatment condition and acclimated for 2.5 min. Then, for the following 2.5 min, the number of times the crab pumped its abdomen was recorded. Immediately following the end of the first crab’s measurement period, another crab was placed into the same treatment to begin its acclimation period. Each crab was returned to a flowing water table between treatments and remained there for at least twenty minutes before beginning the acclimation period of its next treatment. The treatment series began and ended with 30 psu seawater (SW), and proceeded through an increasing gradient of 0, 7, 11, and 17% IRI-160AA in SW + HE.

Each measurement period of the pumping experiments was filmed. The videos were reviewed later, and the time the crabs spent grooming their egg masses was recorded.

A χ^2^ test was performed for the 24 crabs tested to assess if the proportion of crabs performing the behaviors differed among treatments. A one-way repeated-measures ANOVA (Friedman Repeated Measures Analysis of Variance on Ranks) was used to assess trends in the number of pumps and the time spent grooming. Only crabs that performed the behavior were included in each analysis.

### Oyster sub-lethality

#### Respiration

Respiration on oyster pediveligers following methods described for *A. tonsa* nauplii above. Two individuals were placed in each 80 µl well, with rates calculated per individual. Data were analyzed via a one-way ANOVA on Ranks. These experiments all used Batch 3 of IRI-160AA.

#### Activity

Activity experiments on pediveliger larvae were conducted in LAMs and followed similar methods to *Acartia tonsa* and *Callinectes sapidus*. The 24-h data was tested via a one-way ANOVA on ranks, while the data broken down by light phase was analyzed via a one-way repeated measures ANOVA. These experiments used Batch 3 of IRI-160AA.

An additional analysis of pediveliger activity occurred during the mortality experiment by ranking how active each animal appeared to be on a scale of 1 (High Activity, HA, animal was actively swimming), 2 (Medium Activity, MA, animal had its velum extended and cilia active, sometimes scooting across the bottom), 3 (Low Activity, LA, animal was enclosed in its shell but viscera moved when the shell was touched), and 4 (Dead/No Activity, D, animal was completely unresponsive even to repeated stimulation). Ranking occurred at the start of the experiment (where all animals scored as HA), at the 24-h mark, and at the 48-h mark. This assessment was analyzed via a χ^2^ test for both the 24-h and 48-h data sets. At the end of the LAM experiments, animals were analyzed in the same manner.

Activity experiments on the wild-type adult *C. virginica* occurred during the mortality experiments. At each 6-h time point, animals in the containers (0%, 1%, 5%, 10%, 13.5%, 18%, and 24% v/v IRI-160AA treatments) were scored as either Open (O) or Closed (C), and analyzed via a two-way repeated measures ANOVA on the proportion of animals that opened at each time point in each concentration.

#### Feeding

Feeding experiments occurred only on adult *C. virginica*. Animals and containers from the mortality experiments were rinsed to remove algicide residue, then filled with 1 L of 20 psu seawater and *Isochrysis galbana* at ~ 100,000 cells L^−1^, and one animal from each container was returned to it. Five milliliters from each container were removed immediately and in vivo chlorophyll *a* florescence was measured using a fluorometer (Turner Systems). Air stones were added to the containers to keep the algae in suspension, and lids were added to prevent liquid from bubbling out. After 6 h, another fluorescence reading was taken. Animals were given another 6 h to feed, and a final fluorescence reading was taken at the 12-h time point. Clearance rates (CR) were calculated according to Thessen et al.^58^ from time zero to six hours (initial rate, 0–6), and from six to twelve hours (end rate, 6–12), and compared across time ranges and treatments and strains using a three-way ANOVA.

## Supplementary information

Supplementary Information.

## Data Availability

The data generated and analyzed during the current study are available from the corresponding author upon reasonable request.
