# Supplementary material for: Effects of a bacteria-produced algicide on non-target marine invertebrate species
Source: Sci Rep. 2021 Jan 12;11:583. doi: 10.1038/s41598-020-79814-w (PMC7803755; doi:10.1038/s41598-020-79814-w)
Supplement: Supplementary file 1 — Supplementary Information. [file 41598_2020_79814_MOESM1_ESM.docx]

Effects of a bacteria-produced algicide on non-target marine invertebrate species

Victoria E Simons^1^, Kathryn J Coyne^1^, Mark E Warner^1^, Margaret Dolan^1^, Jonathan H Cohen^1*^

^1^University of Delaware, School of Marine Science & Policy, College of Earth, Ocean, and Environment, Lewes, DE 19958, USA

*jhcohen@udel.edu, 302-645-4298


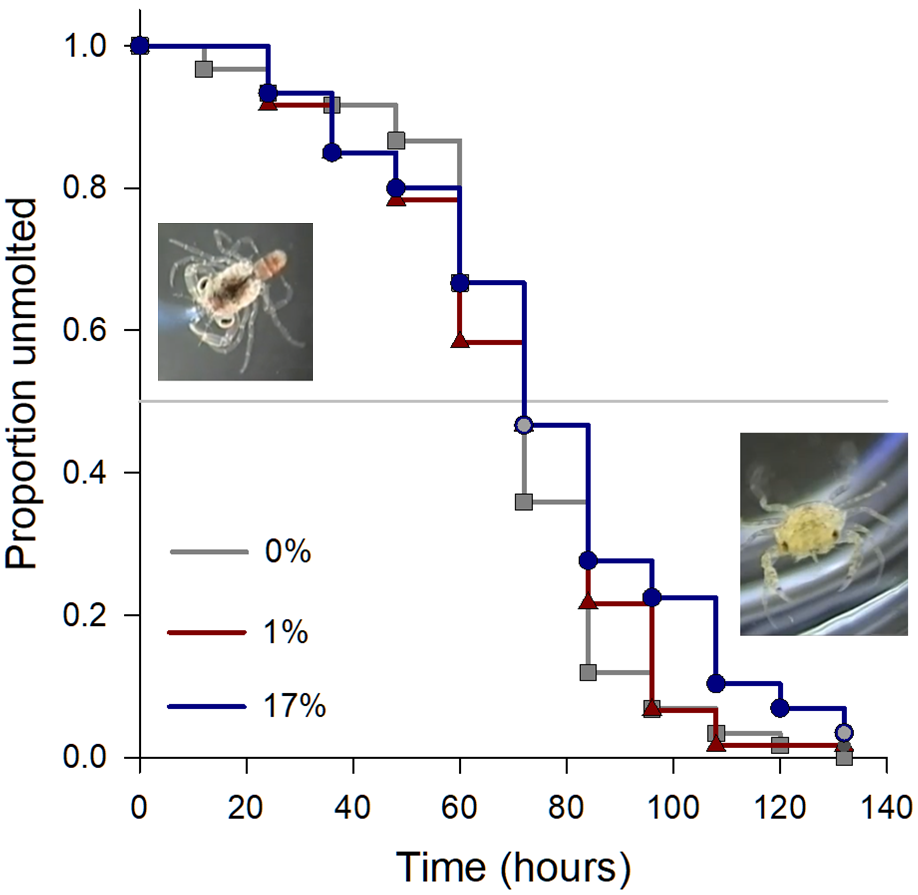


**Supplementary Figure S1.** Molting over time of *C. sapidus* megalopae into first crabs. The light grey line across the middle of the graph marks the 50% molted point.


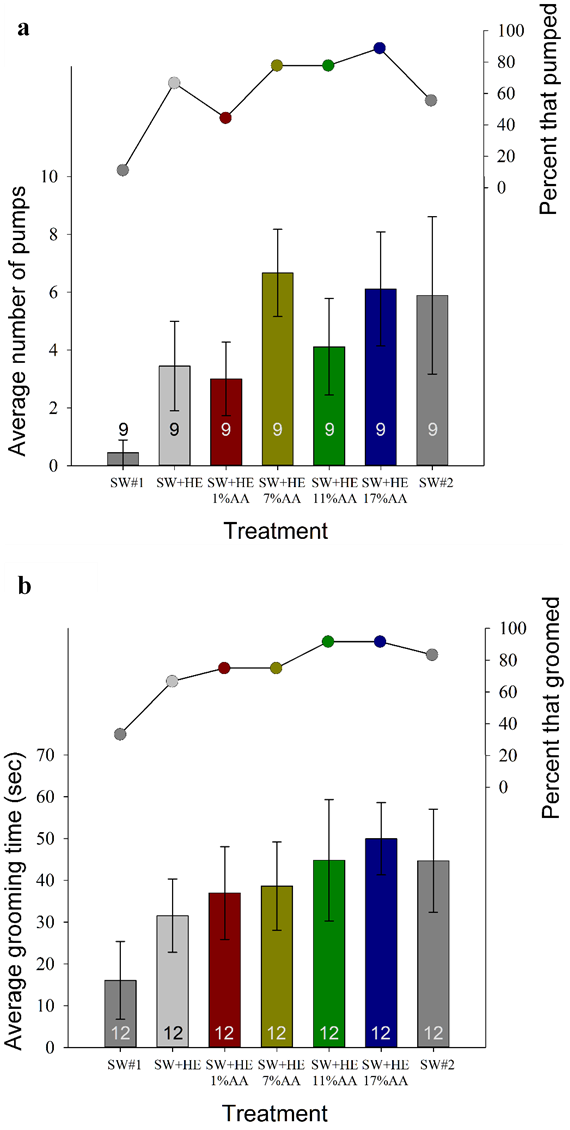


**Supplementary Figure S2.** Pumping and grooming data for *Callinectes sapidus*. Numbers on the bars represent sample size, bars represent standard errors. (a) Pumping rates of *C. sapidus* ovigerous crabs. The line indicates the percentage of crabs that pumped, while the bars represent, out of the crabs that did pump, how many pumps occurred during the 2.5-minute observation period. (b) Grooming behavior of *C. sapidus* ovigerous crabs. The line represents the percent of crabs that groomed at all, while the bars indicate how long the crabs groomed for.


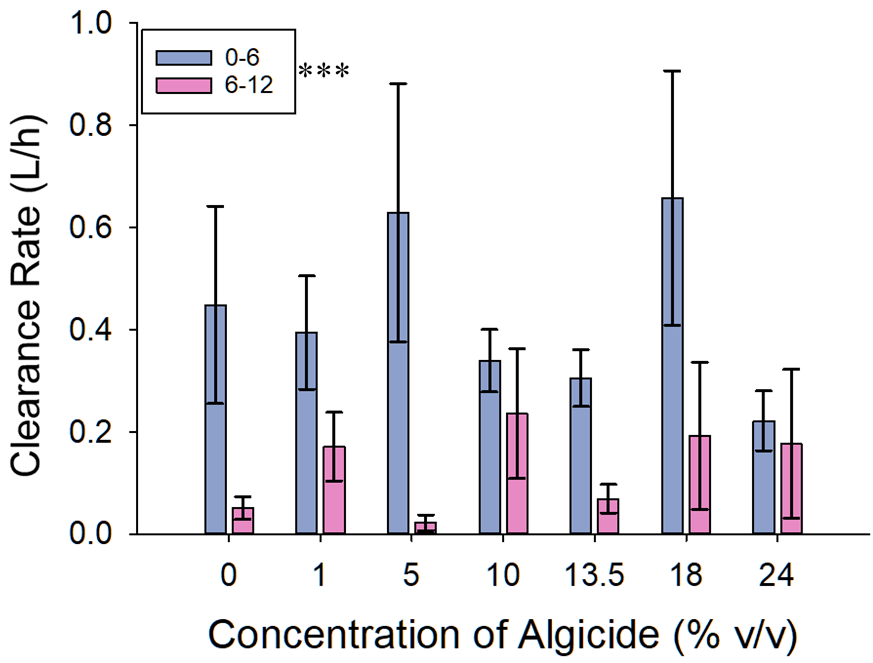


**Supplementary Figure S3.** Clearance rate data for adult *C. virginica*. The initial rate was calculated from 0 to 6 hours and is given in the purple bars, while the end rate was calculated from 6 to 12 hours and is given in the pink bars. There was a significant difference between the two time points: end rates were lower than initial rates overall. (*** = p<0.001)
